# Validity of the total SOFA score in patients ≥ 80 years old acutely admitted to intensive care units: a post-hoc analysis of the VIP2 prospective, international cohort study

**DOI:** 10.1186/s13613-023-01191-0

**Published:** 2023-10-05

**Authors:** Kamil Polok, Jakub Fronczek, Zbigniew Putowski, Marcelina Czok, Bertrand Guidet, Christian Jung, Dylan de Lange, Susannah Leaver, Rui Moreno, Hans Flatten, Wojciech Szczeklik

**Affiliations:** 1 Center for Intensive Care and Perioperative Medicine, Jagiellonian University Medical College, ul. Wrocławska 1-3, 30 – 901, Kraków, Poland; 2 Department of Pulmonology, Jagiellonian University Medical College, Kraków, Poland; 3 Sorbonne Universités, UPMC Univ Paris 06, INSERM, UMR_S 1136, Institut Pierre Louis d’Epidémiologie Et de Santé Publique, Equipe: Epidémiologie Hospitalière Qualité Et Organisation Des Soins, 75012 Paris, France; 4 Assistance Publique-Hôpitaux de Paris, Paris, France; 5 Department of Cardiology, Pulmonology and Vascular Medicine, Medical Faculty, Heinrich-Heine-University Duesseldorf, Moorenstraße 5, 40225 Duesseldorf, Germany; 6 Department of Intensive Care Medicine, University Medical Center, University Utrecht, Utrecht, The Netherlands; 7 Department of Critical Care, St George’s Hospital, London, UK; 8 Hospital de São José, Centro Hospitalar Universitário de Lisboa Central, Faculdade de Ciências Médicas de Lisboa (Nova Médical School), Lisbon, Portugal; 9 Faculdade de Ciências da Saúde, Universidade da Beira Interior, Covilhã, Portugal; 10 Department of Anaesthesia and Intensive Care, Haukeland University Hospital, Bergen, Norway; 11 Department of Clinical Medicine, University of Bergen, Bergen, Norway

## Abstract

**Background:**

Little is known about the performance of the Sequential Organ Failure Assessment (SOFA) score in older critically ill adults. We aimed to evaluate the prognostic impact of physiological disturbances in the six organ systems included in the SOFA score.

**Methods:**

We analysed previously collected data from a prospective cohort study conducted between 2018 and 2019 in 22 countries. Consecutive patients ≥ 80 years old acutely admitted to intensive care units (ICUs) were eligible for inclusion. Patients were followed up for 30 days after admission to the ICU. We used logistic regression to study the association between increasing severity of organ dysfunction and mortality.

**Results:**

The median SOFA score among 3882 analysed patients was equal to 6 (IQR: 4–9). Mortality was equal to 26.1% (95% CI 24.7–27.5%) in the ICU and 38.7% (95% CI 37.1–40.2%) at day 30. Organ failure defined as a SOFA score ≥ 3 was associated with variable adjusted odds ratios (aORs) for ICU mortality dependant on the organ system affected: respiratory, 1.53 (95% CI 1.29–1.81); cardiovascular 1.69 (95% CI 1.43–2.01); hepatic, 1.74 (95% CI 0.97–3.15); renal, 1.87 (95% CI 1.48–2.35); central nervous system, 2.79 (95% CI 2.34–3.33); coagulation, 2.72 (95% CI 1.66–4.48). Modelling consecutive levels of organ dysfunction resulted in aORs equal to 0.57 (95% CI 0.33–1.00) when patients scored 2 points in the cardiovascular system and 1.01 (0.79–1.30) when the cardiovascular SOFA equalled 3.

**Conclusions:**

Different components of the SOFA score have different prognostic implications for older critically ill adults. The cardiovascular component of the SOFA score requires revision.

**Supplementary Information:**

The online version contains supplementary material available at 10.1186/s13613-023-01191-0.

## Background

The Sequential Organ Failure Assessment (SOFA) score, conceived in 1996, was intended to increase our knowledge about organ dysfunction, help us better understand interactions between failing organs, and play a role in the design of clinical trials [1]. Thousands of scientific reports on critically ill patients have incorporated the SOFA score in various ways since then. Despite its popularity, SOFA has been criticised for having failed to reach the goals originally set out for this tool by the Working Group on Sepsis Related Problems of the European Society of Intensive Care Medicine [2]. Accumulating evidence indicates that diagnostic and therapeutic advancements in critical care over the last 30 years have significantly weakened the clinical value of the SOFA score [3].

Simple scoring rules such as SOFA invite the addition of points to arrive at a cumulative score. Notwithstanding the explicit recommendation against the use of the total SOFA as a proxy for the overall severity of multiorgan failure, much of the available literature relies on the total score as a summary measure of organ function [1]. This is problematic as two strong assumptions are necessary for the validity of the total SOFA score: first, each individual organ failure would need to carry the same prognosis; second, categories used by SOFA would have to accurately reflect the degree of organ dysfunction [4].

As is the case for multiple scoring systems in critical care, little is known about the performance of SOFA in older adults. Therefore, we aimed to explore the prognostic impact of physiological disturbances in the six organ systems included in the SOFA score in patients ≥ 80 years old admitted to intensive care units.

## Methods

The Very Old Intensive Care Patient (VIP2) was a prospective, multicentre study conducted in 22 countries and registered on ClinicalTrials.gov (ID: NCT03370692). The study enrolled patients aged 80 years or older acutely admitted to intensive care units (ICUs) without any exclusion criteria. Sources of data, methods of measurement, and results of analyses based on VIP databases were described in detail in the previous paper [5]. Participating ICUs were asked to enrol consecutive patients over a 6-month period with a possibility to end patient accrual after including the 20th participant in the study. A patient’s vital status within 30 days of admission to the ICU was ascertained by inspecting hospital records, direct contact with the patient, or querying a national registry. Participants were recruited between May 2018 and May 2019. Each country had a national coordinator responsible for securing the required ethical and regulatory approvals. A waiver of informed consent for participation in the study was granted in some countries.

Outcomes in this study were ICU- and 30 day mortality. Baseline characteristics included patients’ age, sex, and reason for admission to the ICU. We used the SOFA score to assess the severity of organ dysfunction within the first 24 h after admission to the ICU. Six organ systems are included in the SOFA score: cardiovascular, respiratory, renal, neurologic, hepatic, and coagulation. Between 0 and 4 points were assigned in each organ system, with an increasing number of points corresponding to a more severe organ failure. The highest score observed within the first 24 h was reported. We used the Clinical Frailty Scale (CFS) to describe a patient’s frailty before admission to the hospital, with nine possible classes from very fit prior to the acute illness to terminally ill. Necessary information was given by the patient, their proxy or obtained from the medical records.

Descriptive statistics on baseline variables were presented as medians (interquartile ranges [IQR]) or counts and percentages. The relation between the SOFA score in each organ system and mortality was adjusted for age, sex, reason for admission to the ICU, and the CFS score, which were selected as potential confounders based on the author’s clinical expertise and availability in the dataset. Statistical adjustment was performed using a logistic regression model while keeping age and CFS as continuous variables in distinct models that used either ICU- or 30 day mortality as dependent variables. The SOFA score was modelled twofold: as the original, categorical variable (i.e., 1, 2, 3, or 4 points assigned in each organ system with 0 score as a reference),as a dichotomous indicator of organ failure (i.e., SOFA score ≥ 3 points in each domain), and as a total score, as reported in previous papers. We performed an analogous sensitivity analysis after exclusion of patients in whom life sustaining treatment (LST) was introduced. The required sample size was not calculated a priori. We decided that a complete-case analysis was justified considering the high completeness of data. All analyses were performed using R version 3.6.0 (RProject). Reporting conforms to the STROBE statement [6] (Additional file 1: Table S1).

## Results

**Fig. 1:**
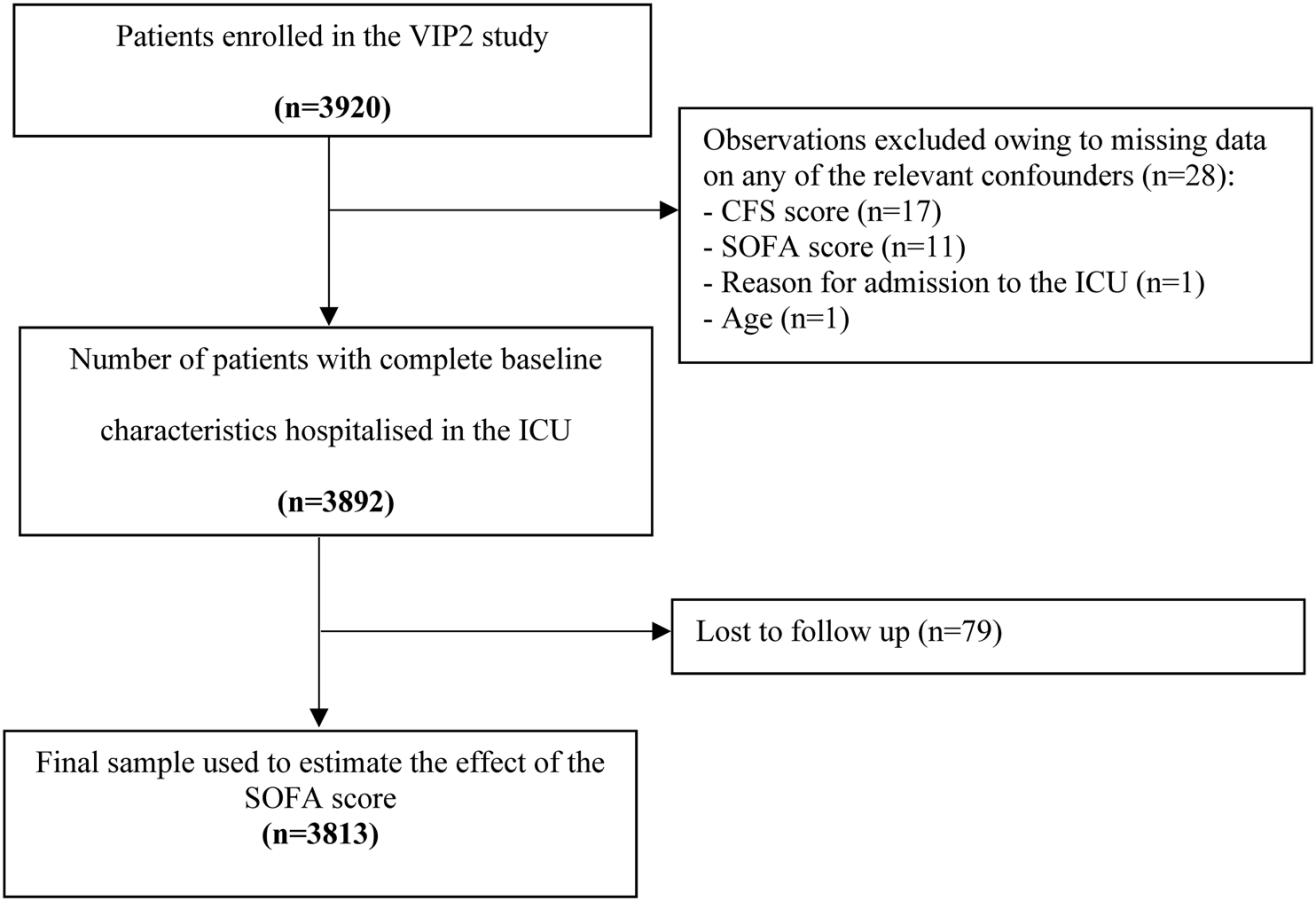
Study flow-chart. CFS, Clinical Frailty Scale; SOFA, Sequential Organ Failure Assessment

**Fig. 2:**
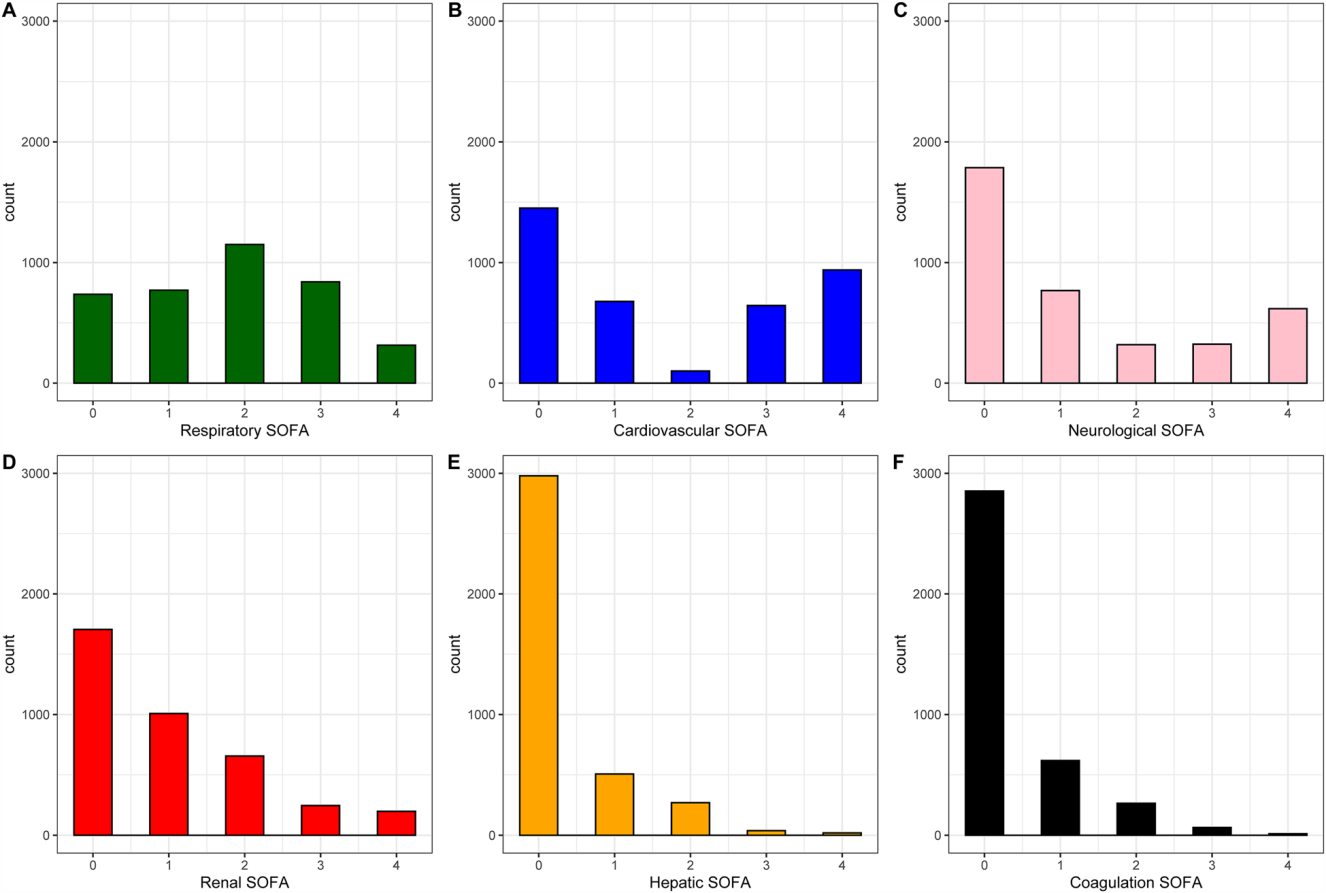
Histograms of the SOFA score by organ system. ICU, intensive care unit; SOFA, Sequential Organ Failure Assessment. **A** Respiratory SOFA, **B** Cardiovascular SOFA, **C** Neurological SOFA, **D** Renal SOFA, **E** Liver SOFA, **F** Coagulation SOFA

Of 3920 patients enrolled in the VIP2 study, 3813 contributed data to analyses of the prognostic impact of the SOFA score (Fig. 1). Patient characteristics were shown in Table 1. Distribution of the SOFA score stratified by organ system was presented in Fig. 2.

**Table 1:** Patient characteristics

Feature	Cohort (n = 3813)
Male gender	2034 (53.3)
Age, years	84 [81 to 87]
Reason for ICU admission	
Emergent surgery	528 (13.8)
Respiratory failure	918 (24.1)
Circulatory failure	524 (13.7)
Combined respiratory & circulatory failure	435 (11.4)
Trauma	228 (6.0)
Neurological	187 (4.9)
Sepsis	525 (13.8)
Other	468 (12.3)
SOFA	6 [4 to 9]
CFS	4 [3 to 6]
Frailty (%)	
Fit	1520 (39.9)
Vulnerable	769 (20.2)
Frail	1524 (40.0)
Non-invasive ventilation	882 (23.2)
Invasive ventilation	1898 (49.8)
Vasopressors	2264 (59.4)
Renal replacement therapy	420 (11.0)
LST withholding	1117 (29.3)
LST withdrawal	535 (14.0)
LST withhold or withdrawal	1304 (34.6)
ICU length of stay	165.93 (232.64)
ICU mortality	1012 (26.5)
30 day mortality	1474 (38.7)

CFS, clinical frailty scale; ICU, intensive care unit; LST, life sustaining treatment, SOFA, sequential organ failure assessment

**Fig. 3:**
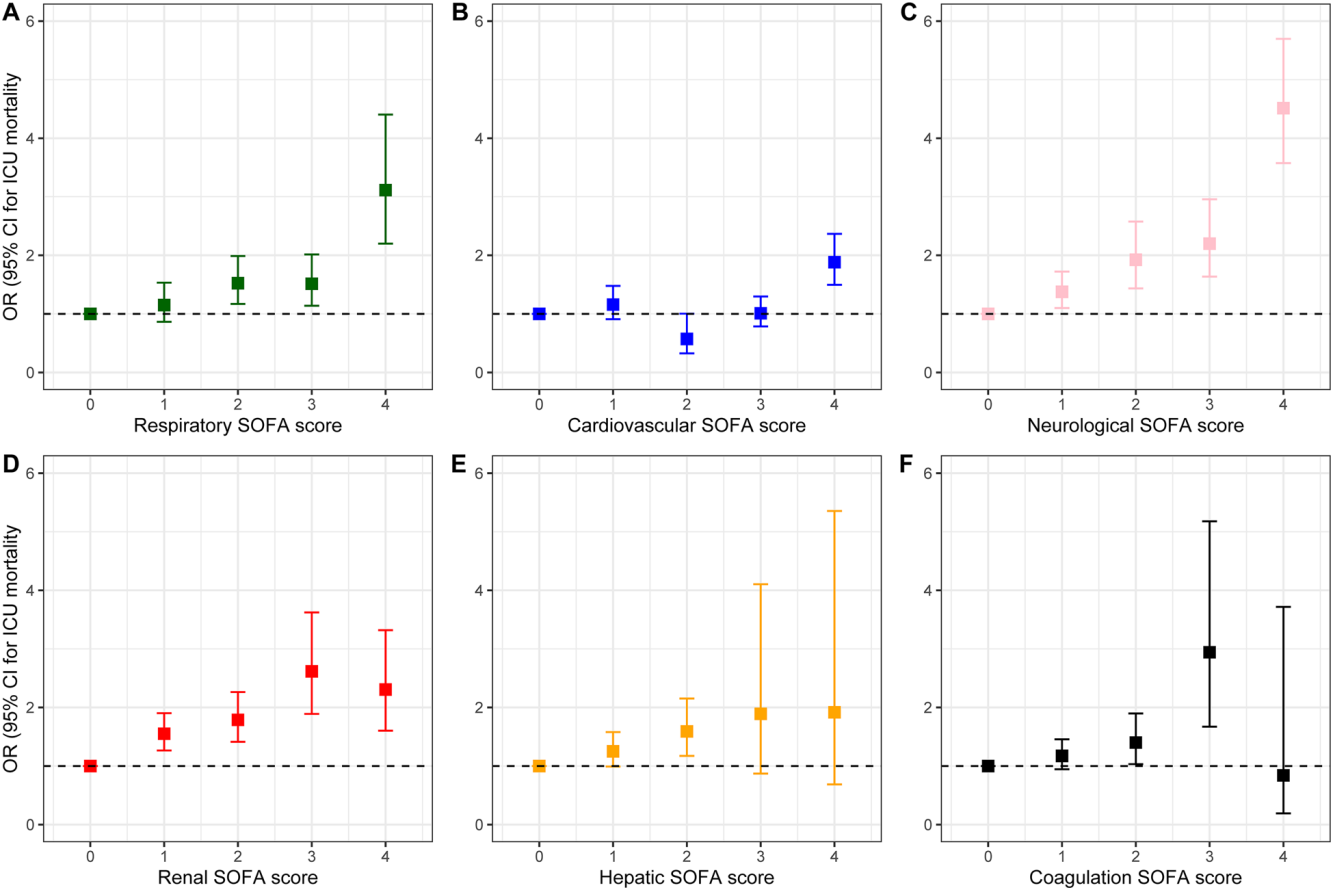
Association between each component of SOFA score with ICU mortality. SOFA, Sequential Organ Failure Assessment. **A** Respiratory SOFA, **B** Cardiovascular SOFA, **C** Neurological SOFA, **D** Renal SOFA, **E** Liver SOFA, **F** Coagulation SOFA

Estimates of both crude and adjusted effects of different organs’ failure on ICU- and 30 day mortality were summarised in Table 2. Organ failure defined as a SOFA score ≥ 3 was associated with variable adjusted odds ratios (aORs) for ICU mortality dependant on the affected organ system: respiratory, 1.53 (95% CI 1.29–1.81); cardiovascular 1.69 (95% CI 1.43–2.01); hepatic, 1.74 (95% CI 0.97–3.15); renal, 1.87 (95% CI 1.48–2.35); central nervous system, 2.79 (95% CI 2.34–3.33); coagulation, 2.72 (95% CI 1.66–4.48). Modelling consecutive levels of organ dysfunction separately resulted in aORs equal to 0.57 (95% CI 0.33–1.00) when patients scored 2 points in the cardiovascular system and 1.01 (0.79–1.30) when the cardiovascular SOFA equalled 3. Adjusted odds ratio for mortality estimated for different categories of the SOFA score were shown in Fig. 3 and Table 3. The total SOFA score was associated with ICU mortality (OR 1.26, 95% CI 1.23 to 1.29) and 30-day mortality (OR 1.20, 95% CI 1.18 to 1.23). Results of the sensitivity analysis including 2468 patients in whom LST limitation was not introduced are summarised in the Additional file 1: Tables S2, 3.

**Table 2:** Logistic regression models, odds ratio for mortality estimated for organ failure (SOFA ≥ 3 in each organ system)

SOFA component	Number of patients with organ failure	ICU mortality		30-day mortality	
		Crude	OR (95% CI)	Crude	OR (95% CI)
Respiratory SOFA	1155 (30.3%)	38.1% vs. 21.5%, p < 0.001	1.53 (1.29 to 1.81)	49.3% vs. 34.0%, p < 0.001	1.36 (1.16 to 1.60)
Cardiovascular SOFA	1583 (41.5%)	37.1% vs. 19.1%, p < 0.001	1.69 (1.43 to 2.01)	49.0% vs. 31.3%, p < 0.001	1.54 (1.32 to 1.80)
Hepatic SOFA	57 (1.5%)	45.6% vs. 26.3%, p = 0.002	1.74 (0.97 to 3.15)	59.6% vs. 38.3%, p = 0.002	1.88 (1.05 to 3.40)
Renal SOFA	444 (11.6%)	38.3% vs. 25.0%, p < 0.001	1.87 (1.48 to 2.35)	50.7% vs. 37.1%, p < 0.001	1.71 (1.37 to 2.13)
Neurological SOFA	940 (24.7%)	47.4% vs. 19.7%, p < 0.001	2.79 (2.34 to 3.33)	58.2% vs. 32.3%, p < 0.001	2.21 (1.87 to 2.62)
Coagulation SOFA	76 (2.0%)	50.0% vs. 26.1%, p < 0.001	2.72 (1.66 to 4.48)	63.2% vs. 38.2%, p < 0.001	2.69 (1.63 to 4.44)

Aside from the SOFA score components the regression model included age, sex, reason for ICU admission and CFS score. Age and CFS score were treated as continuous variables in the model

**Table 3:** Logistic regression models, odds ratio for mortality estimated for original SOFA categories (reference = 0 in each category)

SOFA component	ICU mortality OR (95% CI)	30-day mortality OR (95% CI)
Respiratory		
1	1.15 (0.86 to 1.53)	1.13 (0.89 to 1.44)
2	1.52 (1.17 to 1.99)	1.64 (1.31 to 2.06)
3	1.52 (1.14 to 2.02)	1.48 (1.16 to 1.90)
4	3.11 (2.20 to 4.40)	2.68 (1.94 to 3.70)
Cardiovascular		
1	1.16 (0.91 to 1.48)	1.05 (0.85 to 1.30)
2	0.57 (0.33 to 1.00)	0.53 (033 to 0.87)
3	1.01 (0.79 to 1.30	0.94 (0.75 to 1.17)
4	1.88 (1.50 to 2.37)	1.68 (1.37 to 2.07)
Hepatic		
1	1.25 (0.99 to 1.58)	1.20 (0.97 to 1.48)
2	1.59 (1.17 to 2.15)	1.81 (1.36 to 2.40)
3	1.89 (0.87 to 4.10)	1.96 (0.93 to 4.14)
4	1.92 (0.69 to 5.35)	2.43 (0.86 to 6.87)
Renal		
1	1.55 (1.26 to 1.90)	1.54 (1.28 to 1.84)
2	1.79 (1.41 to 2.26)	1.80 (1.45 to 2.22)
3	2.62 (1.89 to 3.62)	2.54 (1.87 to 3.45)
4	2.31 (1.60 to 3.32)	1.94 (1.39 to 2.71)
Neurological		
1	1.38 (1.10 to 1.72)	1.45 (1.20 to 1.76)
2	1.92 (1.44 to 2.58)	1.61 (1.24 to 2.11)
3	2.20 (1.64 to 2.96)	2.04 (1.55 to 2.68)
4	4.51 (3.58 to 5.70)	3.17 (2.54 to 3.95)
Coagulation		
1	1.17 (0.94 to 1.46)	1.05 (0.87 to 1.28)
2	1.40 (1.03 to 1.90)	1.06 (0.80 to 1.42)
3	2.94 (1.67 to 5.18)	2.45 (1.38 to 4.35)
4	0.84 (0.19 to 3.72)	1.59 (0.45 to 5.62)

Aside from the SOFA score components the regression model included age, sex, reason for ICU admission and CFS score. Age and CFS score were treated as continuous variables in the model

## Discussion

In this multicentre cohort study of patients ≥ 80 years old acutely admitted to ICUs between the years 2018 and 2019, corresponding degrees of organ dysfunction in several organ systems included in the SOFA score translated to substantially different odds of death in the ICU and 30-day observation. Increasing number of points assigned in the cardiovascular component of the SOFA score was not uniformly associated with a poorer prognosis.

Our results corroborate existing evidence of the potential complexity of use of the total SOFA score as a summary measure of multiorgan failure. Pölkki and colleagues have shown that the maximum daily SOFA score measured within the first day after admission to the ICU was not a valid surrogate of mortality in over 60,000 Finnish patients [7]. In their study, the risk of in-hospital death associated with failure of different organs diagnosed using the SOFA score varied widely. Further, the cardiovascular component of the SOFA score did not work as intended due to the rarity and specificity of situations which prompted a dopamine infusion. It is increasingly clear that reliance on dopamine administration as a measure of dysfunction of the cardiovascular system is no longer justifiable, rendering the cardiovascular domain of the SOFA score in need of an urgent revision [8, 9]. This is the effect of a suddenly decreasing role for dopamine in clinical practice e.g. it went from the first choice vasopressor in the 2002 Surviving Sepsis Campaign guidelines to not being mentioned, and being replaced by noradrenaline, vasopressin and epinephrine, in the 2021 update [10]. Recent attempts at creating a unified measure of vasoactive support should facilitate future work on cardiovascular system assessment in the setting of critical illness [2].

Assumptions underlying the use of the total SOFA score may raise some concerns. Equating physiological disturbances in different organ systems in terms of prognosis goes against clinical gestalt and plainly contradicts the current stride towards precision medicine. How does one square the application of sophisticated machine learning algorithms with the use of crude, arbitrary categories to evaluate organ dysfunction? Parameters such as platelet count, PaO_2_/FiO_2_, bilirubin, and creatinine concentration can, and intuitively should, be analysed in a way that respects their continuous nature while maximising the amount of information gained from these measurements [11]. From a clinical point of view, it is also apparent that different combinations of organ dysfunction can have different implications. In the language of statistics, the complex interplay between organ systems can be expressed and properly quantified by employing interaction terms in regression models [12]. Previous studies have convincingly proven that two plus two does not equal four when using the SOFA score, as the relation between the failure of different organs and mortality has a multiplicative rather than additive character [7, 13]. However, one must take some extenuating circumstances into account. First, one of the aspects that made SOFA score so popular is its simplicity and the ability to evaluate it at the bedside. Second, the introduction of a complex clinical tool using machine learning, assessment of statistical interactions and other sophisticated mathematical tools would not have been feasible in 1996. Conversely, the current availability of smartphones, much more powerful than personal computers means there is great potential for the creation of new prognostic tools and this should be considered when the decision is made to update the SOFA score [14].

The SOFA score has permeated critical care [15, 16]. Diagnostic criteria of sepsis are now based on a change in the SOFA score [17]. However, if one were to take a step back, the literature begs the following question: do we really need a numerical score to describe organ failure? Even if this is the case, a reliable score would have to be organ-specific (1), independent of therapy (2), reflect acute dysfunction that does not overlap with chronic dysfunction (3) and be reproducible in heterogeneous groups of ICU patients (4) [4]. The results of our study do not support the above conditions in regards to the SOFA score in older patients. The seminal consensus indicates that SOFA should be able to broaden our knowledge about organ failure and facilitate the conduct of clinical trials. On the one hand, a meta-regression of 58 randomized controlled trials showed that SOFA score measured at one time-point is not an optimal surrogate for mortality [11]. Based on our results, we know that despite its significant association with mortality, total SOFA score fails to reliably describe multiorgan failure in the older population. On the other hand, delta of SOFA score is well associated with mortality in randomised controlled trial. Unfortunately, in this study we only gathered the worst SOFA score within 24 h of ICU admission and therefore we are unable to determine whether assessment of SOFA score trends translates better into mortality than its single measurement.

For the past decades, experts in research methods and statistics have repeatedly reminded our community that arbitrary categorisation of data is a waste at best and can lead to harm in the worst-case scenario [18, 19]. If a pattern of physiological parameters (i.e., sequential organ failure assessment) is of interest, nothing stands in the way of plotting raw clinical and laboratory measurements over time and analysing them in their original form. Data will speak for themselves and reveal both the strength and complexity of estimated effects if nonlinearities such as U-shaped relations are allowed at the stage of statistical analysis. Even though the European Medicines Agency encouraged trialists to use the SOFA score as an endpoint, the SOFA score’s capacity to explain mortality, estimated at ≤ 35% when using the delta SOFA score, is far below the 85% bar set by the Food and Drug Administration for surrogate outcomes in oncology [11, 20]. New patient-oriented outcomes, such as days alive and free from organ support or days alive outside the ICU within a predefined period, such as 28 or 90 days, have recently gained popularity. These endpoints have far more promising properties than arbitrary categories of uncertain importance to patients [21, 22]. Better still, longitudinal ordinal models can be used to incorporate all relevant transitions between stages of critical illness and maximise statistical power, though these models require a relatively high level of expertise and effort from the study’s biostatistician [23].

This study has several weaknesses. First, only the maximum SOFA score within the first 24 h after admission to the ICU was available in our dataset, precluding any exploration of changes in the SOFA score over time and their relations with mortality. We also did not record the baseline SOFA score, which would provide us with valuable information of chronic organ failure in the population of older critically ill patients. Importantly, the dynamics of each of the SOFA components express different time trajectories during the ICU hospitalisation. Nevertheless, limitations described above apply to any transformation of the original score. Second, the sample size did not allow for a credible investigation of interactions between different systems and subgroups based on the reason for admission to the ICU. It also led to low number of patients and wide confidence intervals in the analysis of the highest categories in renal, hepatic and coagulation components, potentially resulting in some difficulties in interpretation of the results. Third, we did not assess the interrater variability, we know that parameters such as the Glasgow Coma Scale are prone to misclassification in critically ill patients. Fourth, raw data such as biomarker concentrations were not collected in the primary study. Fifth, this is a post-hoc analysis and our results should be considered hypothesis-generating. Sixth, these results are generalisable primarily to older adults. Further studies developed by the VIP project group will help to address this issue more precisely in the future, however an optimal way to assess SOFA score performance in the population of older ICU patients would be to design a large prospective study focused on SOFA score validation on a dedicated cohort.

Our study has many strengths. We were able to include almost four thousand patients ≥ 80 years old from an international, prospectively enrolled cohort, and the completeness of relevant data was exceptionally high. Outcomes included both ICU- and 30-day mortality, mitigating the risk of biases arising from hospital wards’ discharge policies. Our study finished recruitment before the COVID-19 pandemic, thus our results are applicable to a broad population of older patients routinely treated in intensive care units.

## Conclusion

Different components of the SOFA score have different prognostic implications for older critically ill adults. The cardiovascular component of the SOFA score needs revision. Future research should explicitly test the utility of the SOFA score with reference to other methods of organ function assessment.

### Supplementary Information

**Additional file 1: Table S1.** STROBE Statement—checklist of items that should be included in reports of observational studies. **Table S2.** Logistic regression models, odds ratio for mortality estimated for organ failure (SOFA ≥ 3 in each organ system) in the sensitivity analysis including patients without LST limitation. **Table S3.** Logistic regression models, odds ratio for mortality estimated for original SOFA categories (reference = 0 in each category) in the sensitivity analysis including patients without LST limitation.

## Data Availability

The datasets used and/or analysed during the current study are available from the corresponding author on reasonable request.

## Abbreviations

CFS: Clinical Frailty Scale
ICU: Intensive Care Unit
SOFA: Sequential Organ Failure Assessment
