# Supplementary material for: Validity of the total SOFA score in patients ≥ 80 years old acutely admitted to intensive care units: a post-hoc analysis of the VIP2 prospective, international cohort study
Source: Ann Intensive Care. 2023 Oct 5;13:98. doi: 10.1186/s13613-023-01191-0 (PMC10555975; doi:10.1186/s13613-023-01191-0)
Supplement: Supplementary file 1 — Additional file 1: Table S1. STROBE Statement—checklist of items that should be included in reports of observational studies. Table S2. Logistic regression models, odds ratio for mortality estimated for organ failure (SOFA ≥ 3 in each organ system) in the sensitivity analysis including patients without LST limitation. Table S3. Logistic regression models, odds ratio for mortality estimated for original SOFA categories (reference = 0 in each category) in the sensitivity analysis including patients without LST limitation. [file 13613_2023_1191_MOESM1_ESM.docx]

**SUPPLEMENTARY MATERIAL**

**Title:** Validity of the total SOFA score in patients ≥80 years old acutely admitted to intensive care units: a post-hoc analysis of the VIP2 prospective, international cohort study.

**Authors:** Kamil Polok^1,2^, Jakub Fronczek^1^, Zbigniew Putowski^1^, Marcelina Czok^1^, Bertrand Guidet^3^, Christian Jung^4^, Dylan de Lange^5^, Susannah Leaver^6^, Rui Moreno^7^, Hans Flatten^8,9^, Wojciech Szczeklik^1^

**List of contents:**

**Supplementary Table 1.** STROBE Statement—checklist of items that should be included in reports of observational studies

**Supplementary Table 2.**  Logistic regression models, odds ratio for mortality estimated for organ failure (SOFA ≥3 in each organ system) in the sensitivity analysis including patients without LST limitation.

**Supplementary Table 3.** Logistic regression models, odds ratio for mortality estimated for original SOFA categories (reference = 0 in each category) in the sensitivity analysis including patients without LST limitation.

**Supplementary Table 1.** STROBE Statement—checklist of items that should be included in reports of observational studies

|  | Item No. | Recommendation | Page  No. |
| --- | --- | --- | --- |
| **Title and abstract** | 1 | (*a*) Indicate the study’s design with a commonly used term in the title or the abstract | 1 |
|  |  | (*b*) Provide in the abstract an informative and balanced summary of what was done and what was found | 2 |
| Introduction | | | |
| Background/rationale | 2 | Explain the scientific background and rationale for the investigation being reported | 4 |
| Objectives | 3 | State specific objectives, including any prespecified hypotheses | 4 |
| Methods | | | |
| Study design | 4 | Present key elements of study design early in the paper | 4-5 |
| Setting | 5 | Describe the setting, locations, and relevant dates, including periods of recruitment, exposure, follow-up, and data collection | 5 |
| Participants | 6 | (*a*) *Cohort study*—Give the eligibility criteria, and the sources and methods of selection of participants. Describe methods of follow-up  *Case-control study*—Give the eligibility criteria, and the sources and methods of case ascertainment and control selection. Give the rationale for the choice of cases and controls  *Cross-sectional study*—Give the eligibility criteria, and the sources and methods of selection of participants | 5 |
|  |  | (*b*) *Cohort study*—For matched studies, give matching criteria and number of exposed and unexposed  *Case-control study*—For matched studies, give matching criteria and the number of controls per case | NA |
| Variables | 7 | Clearly define all outcomes, exposures, predictors, potential confounders, and effect modifiers. Give diagnostic criteria, if applicable | 5 |
| Data sources/ measurement | 8* | For each variable of interest, give sources of data and details of methods of assessment (measurement). Describe comparability of assessment methods if there is more than one group |  |
| Bias | 9 | Describe any efforts to address potential sources of bias | 5-6 |
| Study size | 10 | Explain how the study size was arrived at | 6 |

| Quantitative variables | | 11 | | Explain how quantitative variables were handled in the analyses. If applicable, describe which groupings were chosen and why | *5-6* |  |
| --- | --- | --- | --- | --- | --- | --- |
| Statistical methods | | 12 | | (*a*) Describe all statistical methods, including those used to control for confounding | 5-6 |  |
|  |  |  |  | (*b*) Describe any methods used to examine subgroups and interactions | 5-6 |  |
|  |  |  |  | (*c*) Explain how missing data were addressed | 5-6 |  |
|  |  |  |  | (*d*) *Cohort study*—If applicable, explain how loss to follow-up was addressed  *Case-control study*—If applicable, explain how matching of cases and controls was addressed  *Cross-sectional study*—If applicable, describe analytical methods taking account of sampling strategy | 5-6 |  |
|  |  |  |  | (*e*) Describe any sensitivity analyses | NA |  |
| Results | | | | | | |
| Participants | | 13* | | (a) Report numbers of individuals at each stage of study—eg numbers potentially eligible, examined for eligibility, confirmed eligible, included in the study, completing follow-up, and analysed | 6 |  |
|  |  |  |  | (b) Give reasons for non-participation at each stage | F1 |  |
|  |  |  |  | (c) Consider use of a flow diagram | F1 |  |
| Descriptive data | | 14* | | (a) Give characteristics of study participants (eg demographic, clinical, social) and information on exposures and potential confounders | T1 |  |
|  |  |  |  | (b) Indicate number of participants with missing data for each variable of interest | F1 |  |
|  |  |  |  | (c) *Cohort study*—Summarise follow-up time (eg, average and total amount) | NA |  |
| Outcome data | | 15* | | *Cohort study*—Report numbers of outcome events or summary measures over time | *6* |  |
|  |  |  |  | *Case-control study—*Report numbers in each exposure category, or summary measures of exposure |  |  |
|  |  |  |  | *Cross-sectional study—*Report numbers of outcome events or summary measures |  |  |
| Main results | | 16 | | (*a*) Give unadjusted estimates and, if applicable, confounder-adjusted estimates and their precision (eg, 95% confidence interval). Make clear which confounders were adjusted for and why they were included | 6, T2-3 |  |
|  |  |  |  | (*b*) Report category boundaries when continuous variables were categorized | NA |  |
|  |  |  |  | (*c*) If relevant, consider translating estimates of relative risk into absolute risk for a meaningful time period | NA |  |
| Other analyses | 17 | | Report other analyses done—eg analyses of subgroups and interactions, and sensitivity analyses | | NA |  |
| Discussion | | | | | | |
| Key results | 18 | | Summarise key results with reference to study objectives | | 6-7 |  |
| Limitations | 19 | | Discuss limitations of the study, taking into account sources of potential bias or imprecision. Discuss both direction and magnitude of any potential bias | | 9-10 |  |
| Interpretation | 20 | | Give a cautious overall interpretation of results considering objectives, limitations, multiplicity of analyses, results from similar studies, and other relevant evidence | | 9-10 |  |
| Generalisability | 21 | | Discuss the generalisability (external validity) of the study results | | 9-10 |  |
| Other information | | |  | | | |
| Funding | 22 | | Give the source of funding and the role of the funders for the present study and, if applicable, for the original study on which the present article is based | | 11 |  |

**Supplementary Table 2.** Logistic regression models, odds ratio for mortality estimated for organ failure (SOFA ≥3 in each organ system) in the sensitivity analysis including patients without LST limitation.

| **SOFA component** | **ICU mortality**  **OR (95% CI)** | **30-day mortality**  **OR (95% CI)** |
| --- | --- | --- |
| Respiratory SOFA | 1.71  (1.29 to 2.26) | 1.35  (1.07 to 1.70) |
| Cardiovascular SOFA | 2.43  (1.83 to 3.23) | 1.75  (1.40 to 2.20) |
| Hepatic SOFA | 1.39  (0.55 to 3.55) | 1.43  (0.62 to 3.27) |
| Renal SOFA | 2.55  (1.78 to 3.63) | 1.96  (1.44 to 2.65) |
| Neurological SOFA | 2.71  (2.04 to 3.58) | 1.95  (1.53 to 2.48) |
| Coagulation SOFA | 1.71  (1.29 to 2.26) | 2.75  (1.26 to 5.96) |

**Footnote:** Aside from the SOFA score components the regression model included age, sex, reason for ICU admission and CFS score. Age and CFS score were treated as continuous variables in the model.

**Supplementary Table 3.** Logistic regression models, odds ratio for mortality estimated for original SOFA categories (reference = 0 in each category) in the sensitivity analysis including patients without LST limitation.

| **SOFA component** | **ICU mortality**  **OR (95% CI)** | **30-day mortality**  **OR (95% CI)** |
| --- | --- | --- |
| Respiratory |  |  |
| 1 | 1.30 (0.79 to 2.14) | 1.25 (0.87 to 1.80) |
| 2 | 1.48 (0.92 to 2.37) | 1.76 (1.25 to 2.48) |
| 3 | 1.64 (1.00 to 2.68) | 1.58 (1.09 to 2.30) |
| 4 | 3.33 (1.86 to 5.95) | 2.57 (1.60 to 4.13) |
| Cardiovascular |  |  |
| 1 | 1.08 (0.69 to 1.68) | 0.91 (0.66 to 1.26) |
| 2 | 0.46 (0.19 to 1.14) | 0.46 (0.23 to 0.91) |
| 3 | 1.46 (0.97 to 2.20) | 1.11 (0.81 to 1.53) |
| 4 | 2.27 (1.54 to 3.35) | 1.61 (1.18 to 2.18) |
| Liver |  |  |
| 1 | 1.31 (0.91 to 1.90) | 1.15 (0.85 to 1.55) |
| 2 | 2.46 (1.58 to 3.84) | 2.63 (1.81 to 3.81) |
| 3 | 1.79 (0.48 to 6.62) | 1.45 (0.46 to 4.60) |
| 4 | 1.69 (0.41 to 6.92) | 2.33 (0.66 to 8.20) |
| Renal |  |  |
| 1 | 2.24 (1.59 to 3.16) | 1.88 (1.45 to 2.45) |
| 2 | 2.23 (1.50 to 3.32) | 1.87 (1.36 to 2.56) |
| 3 | 4.37 (2.62 to 7.31) | 3.49 (2.28 to 5.43) |
| 4 | 4.24 (2.40 to 7.48) | 2.20 (1.36 to 3.57) |
| Neurological |  |  |
| 1 | 1.50 (1.01 to 2.22) | 1.35 (1.01 to 1.81) |
| 2 | 2.54 (1.61 to 4.00) | 1.79 (1.24 to 2.60) |
| 3 | 3.38 (2.13 to 5.37) | 2.36 (1.61 to 3.45) |
| 4 | 4.57 (3.09 to 6.77) | 2.56 (1.86 to 3.54) |
| Coagulation |  |  |
| 1 | 1.67 (1.21 to 2.32) | 1.22 (0.93 to 1.60) |
| 2 | 1.53 (0.94 to 2.47) | 1.11 (0.74 to 1.68) |
| 3 | 4.79 (1.75 to 13.12) | 2.39 (0.94 to 6.08) |
| 4 | 2.83 (0.39 to 20.38) | 2.75 (0.45 to 16.80) |

**Footnote:** Aside from the SOFA score components the regression model included age, sex, reason for ICU admission and CFS score. Age and CFS score were treated as continuous variables in the model.
